# Effect of the association of continuous shortwave diathermy and Pilates-based exercises on pain, depression, and anxiety in chronic non-specific low back pain: a randomized clinical trial

**DOI:** 10.1590/1414-431X2023e12338

**Published:** 2023-03-17

**Authors:** S. Amaral, A.C. Pássaro, R.A. Casarotto

**Affiliations:** 1 Departamento de Fisioterapia, Fonoaudiologia, e Terapia Ocupacional, Faculdade de Medicina, Universidade de São Paulo, São Paulo, SP, Brasil

**Keywords:** Shortwave therapy, Exercise and movement techniques, Backache, Randomized controlled clinical trial, Physiotherapy

## Abstract

Chronic nonspecific low back pain (CNLBP) is the most common musculoskeletal condition, which can be influenced by nociceptive, psychosocial, cognitive, and affective aspects, causing vulnerabilities and impairing the individual's ability to manage pain. The association of continuous shortwave diathermy (CSWD) with Pilates-based exercises may contribute to reduce pain, depression, and anxiety in patients with CNLBP. A single-blind randomized clinical trial was performed in which 36 patients with CNLBP were divided into a control group that received placebo CSWD and an intervention group that received active CSWD. Both groups received 12 sessions of Pilates-based exercises. Pain, depression, and anxiety variables were evaluated using the McGill questionnaire, the Beck Depression Inventory, and the Visual Analog Anxiety Scale. Assessments were performed at baseline, after three and six weeks of treatment, and at the three-month follow-up. The Shapiro-Wilk test, Student's *t*-test, Mann-Whitney U test, chi-squared test, and repeated measures ANOVA, with α=0.05, were used to compare the outcomes, and indicated that active CSWD did not present additional improvement in the assessed variables in CNLBP patients compared to the placebo group. Both groups improved pain and depression at follow-up and reduced anxiety only during Pilates-based exercises. Therefore, only Pilates-based exercises seemed sufficient to manage patients with CNLBP.

## Introduction

Low back pain (LBP) is the world's most prevalent and disabling condition and is generally classified according to the duration of pain in acute (<6 weeks), subacute (6-12 weeks), and chronic (>12 weeks) (1). LBP poses substantial challenges for the health system and socioeconomic status, affecting approximately 25% of working-age adults worldwide (2).

Data related to LBP have predominantly been published in high-income countries (3). Research shows that the number of people with disabilities caused by chronic nonspecific LBP (CNLBP) has increased by 54% in the last 30 years, with the total cost of treatment in the United States estimated to exceed US$ 100 billion per year (4). South Asia alone recorded 10.8 million years lived with disability in 2017 and Southern Latin America had the highest age-standardized years lived with disability rate (1404 per 100,000 persons) in that year (3).

A specific cause of LBP is rarely identified (pain and other symptoms caused by specific pathophysiological mechanisms of nonspinal or spinal origin); hence, most LBPs are termed nonspecific (back pain [with or without leg pain] without a clear nociceptive specific cause). CNLBP has physical risk factors, such as prolonged standing or walking, lifting heavy weights, and psychological factors, such as depression and job dissatisfaction. High levels of depression or anxiety are consistently associated with poor outcomes (i.e., ongoing pain and/or disability) in patients with LBP (1,5).

Recently, studies have reported that comorbidities of depression-anxiety-chronic pain-insomnia by neurosensitization processes are similar to those of epilepsy, in which a persistent increase in neuronal reactivity occurs. Neurosensitization is the basis of a common etiology of chronic pain, depression, and anxiety disorders and can cause clinical manifestations to be increasingly spontaneous, persistent, and severe (6).

According to the existing literature, non-pharmacological treatment of CNLBP involves interventions to help people manage health conditions effectively and maintain quality of life because it is a potential strategy to reduce pain-related disability (self-management support) (7). Moreover, exercise has been recommended, with moderate levels of evidence in the Guidelines of the American College of Physicians (8), and deep heat treatment via shortwave diathermy (SWD) has been effective in reducing pain (9,10).

Pilates and segmental stabilization of the spine aim to activate the deep and stabilizing muscles of the trunk, that is, muscles directly connected to the vertebrae and responsible for segmental spinal control, to stabilize the trunk and limb movements, thus positively impacting the management of CNLBP (1,11).

A network meta-analysis by Hayden et al. (12) pointed out that Pilates, compared to minimal treatment, other effective treatments, and other types of exercises, produced moderate to clinically significant treatment effects, making Pilates an interesting choice for the treatment of CNLBP (13). Fleming et al. (14) found positive effects of acute Pilates training, such as a significant reduction in state anxiety, feelings of fatigue, and overall mood disturbance among young adult male patients. Aibar-Almazán et al. (15) analyzed the effects of a Pilates exercise program in women and found beneficial effects on sleep quality, anxiety, depression, and fatigue. A study by Farzane and Koushkie Jahromi (16) suggested that Pilates training could improve mental and physical function and decrease depression (67%) and anxiety (53%). One possible explanation is that Pilates increases β-endorphin levels (17), which in turn has antidepressant effects (18).

Therapeutic heat has also been used as a non-pharmacological treatment to decrease pain in patients (19); therefore, the use of a thermotherapeutic agent, such as SWD-associated Pilates, could provide additional improvement in the management of CNLBP. Kerem and Yigiter (20) investigated the effects of pulsed (200 and 46 Hz) and continuous SWD on LBP with root irritation. The three groups showed improvement in pain, muscle strength, and range of motion, with better results in the pulsed SWD group. This result is probably due to the fact that the authors treated an acute condition such as root irritation, which responds better to thermal therapy such as pulsed SWD (21).

The generation of intense heat in the body activates transient receptor potential vanilloid (TRPV1) receptors, which are abundantly expressed in the limbic system and the systemic nuclei involved in emotion management (22). The activation of these heat-triggered receptors may contribute to the modulation of depression (23).

Other studies have also suggested that active SWD associated with an exercise program is effective for CNLBP (10,24). Despite being widely used in physiotherapy clinical practice, to our knowledge, no randomized clinical trials have been conducted following the recommendations of the Consolidated Standards of Reporting Clinical Trials statement (CONSORT) and SWD-associated Pilates in patients with CNLBP.

A recent systematic review on the use of SWD in chronic LBP revealed that studies lacked content; additionally, existing studies have low methodological quality with undeclared study designs and inadequate descriptions of the parameters used. The authors of the systematic review also pointed to the need for new randomized clinical trials on this subject with a special focus on detailing the prescription parameters and methodological rigor used in SWD (25).

Therefore, this study aimed to evaluate the effect of the association between CSWD and Pilates on reducing pain, depression, and anxiety in patients with CNLBP. We hypothesized that this association is beneficial for decreasing pain, depression, and anxiety in these patients.

## Material and Methods

### Study design

**Figure 1:**
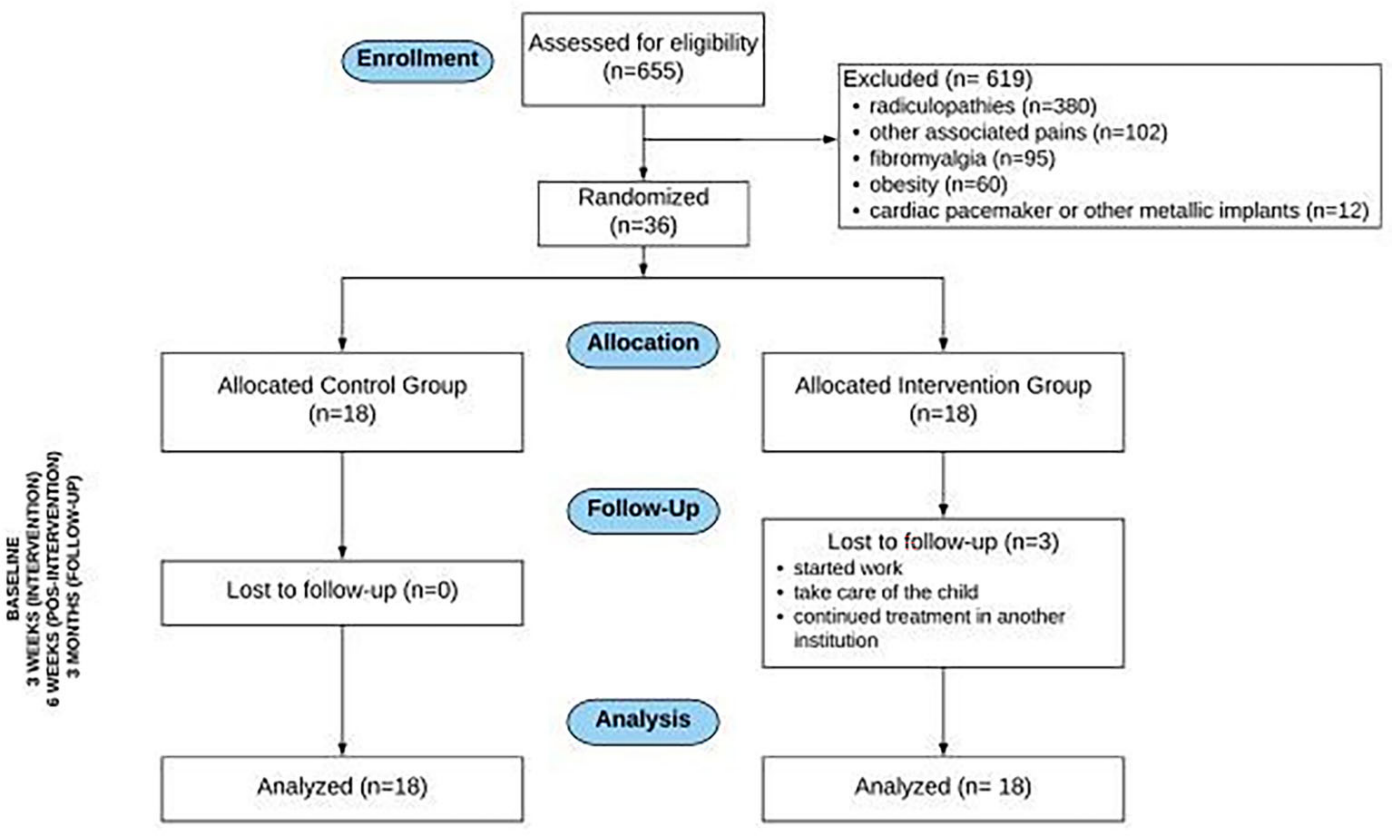
Consort flowchart for enrollment, randomization, and treatment of the participants. Intervention Group: completed cases using the intention-to-treat analysis.

This single-blinded (evaluator) randomized controlled clinical trial was conducted at the Laboratory of Clinical Physiotherapeutic Investigation and Electroneuromyography, Department of Speech Therapy, Physiotherapy, and Occupational Therapy, Faculty of Medicine of the University of São Paulo from August 2016 to August 2019. The therapist who administered the treatment was not blinded to the groups. Randomization was performed by a researcher who was not involved in data collection using a website (www.randomization.com). Numbers 1 to 36 were randomly assigned to each participant after the initial evaluation in opaque and sealed envelopes. Patients were blinded about the groups and recruited from the orthopedic department of the Hospital of University of São Paulo (suitable students, professors, and employees were enrolled). All participants were diagnosed with CNLBP by doctors from different sectors of the public or private service. CNLBP was diagnosed after excluding specific disorders of spinal and non-spinal origin. It is typically defined as pain below the costal margin and above the inferior gluteal folds (>12 weeks), with or without leg pain, without a clear specific nociceptive cause such as disk herniation, spinal stenosis, fracture, tumor, infection, and axial spondyloarthritis. Diagnosis is usually made through history and physical examination. Imaging tests are not routinely indicated in patients with nonspecific low back pain (1) (Figure 1).

The inclusion criteria were CNLBP for ≥3 months, no history of irradiation to the lower limbs, pain intensity ≥3 points on the 10-point pain scale, pain frequency of at least three times a week, age 18-72 years, both sexes, and ability to attend the sessions.

Exclusion criteria were body mass index >30 kg/m^2^, previous spine surgeries with metallic implants, severe spinal disease (tumors, infection, non-union or malunion fractures, inflammatory diseases, and radiculopathies), rheumatic disease, degenerative or inflammatory diseases, acute nonspecific LBP, ongoing physical therapy or drug treatments for musculoskeletal pain, depression, or anxiety, pregnancy, contraindication to performing physical activity according to the American College of Sports Medicine guidelines (7), and current labor dispute, which can be a factor that reinforces the chronicity of LBP due to the economic gains and social benefits derived from the disease (26).

Treatments were delivered by the researcher twice a week, totaling 12 sessions of 1 h for each patient. Patients were instructed not to participate in any other treatment during the study period, including physical activity, and inform if they needed medication for musculoskeletal pain, depression, and anxiety.

Patients were divided into two groups of 18 participants each: the intervention group, which received 20 min of continuous SWD application and the control group, which received placebo SWD, that is, they were positioned similarly to the intervention participants, with the plates maintained under the lumbar region for 20 min with the equipment in off mode.

The equipment used was shortwave diathermy (THERMOWAVE^®^, BIOSET, Brazil) with an emission frequency of 27.12 MHz and a continuous mode of 180 W (+/-30%). The application was performed with the patient in the supine position, with two plate-type electrodes wrapped in a towel to absorb moisture using the coplanar application method in parallel vertically placed on the right and left sides of the lumbar region, with a distance of 5-10 cm between them (19). In the intervention group, the heat intensity was adjusted according to patient reference for pleasant warmth without burning sensation.

For both groups, the Pilates protocol was performed for 40 min (mat Pilates modality), totaling 12 exercises with 15 slow repetitions each and a rest interval of three to five breaths. All exercises were performed individually and were supervised by the researcher (14,15,27). The exercises executed were:

1. Awareness and activation of the pelvic floor muscles and abdominal core: a) Neutral Hip Position: dorsal decubitus with knees flexed and feet supported with contraction of the abdomen and pelvic floor; b) Rail Abdominal: dorsal decubitus with extended hips and legs and trunk elevation.
2. Engagement of the arm muscles: a) Hundred: dorsal decubitus with knees flexed, feet supported, and arm swing.
3. Stretching and strengthening of the flexor and extensor muscles of the ankles: a) Leg Stretch with Foot Movement: dorsal decubitus with one leg raised with foot flexion and extension, and the other flexed with foot supported.
4. Strengthening of the extensor muscles of the knees: a) Single Leg Stretch I: dorsal decubitus with raised leg and knee flexion and extension, and the other knee flexed with foot supported.
5. Strengthening of the extensor and flexor muscles of the hips: a) Bridge: dorsal decubitus with knees flexed, feet supported, and hips elevated; b) Single table top: dorsal decubitus raising and lowering one leg flexed at 90 degrees, and the other with knee flexed and foot supported; c) Single leg stretch II: dorsal decubitus raising and lowering one leg outstretched, and the other with knee flexed and foot supported.
6. Strengthening of the adductor and abductor muscles of the hip: a) Oyster Frontal: dorsal decubitus opening and closing knees with feet supported; b) Lateral Oyster: lateral decubitus with flexed knees, moving the top knee apart and closer.
7. Stretching of the spine: a) Hug Legs: dorsal decubitus hugging bent legs; b) Dog Stretch: four supports with glutes in the heels and spine elongated downwards.

Pilates emphasizes strengthening and activation of the deep muscles of the trunk, posterolateral and anteromedial chains of the hips and thighs, sacroiliac stabilization, flexibility of the lower limbs and spinal muscles, and overall muscles resistance (28,29).

All participants were instructed on appropriate postures during sleep, activities of daily living, and work. These guidelines were followed for both groups during the initial three sessions of SWD (30). The study was approved by the Ethics Committee of FMUSP (CAAE 48391615.9.0000.0065/Opinion Number: 1.205.134) and registered in the Clinical Trials Platform Trial Registration (NCT04048902). All the participants signed a free and informed consent form.

### Sensory and affective aspects of pain

The Brazilian version of the McGill Pain Questionnaire contains 67 words describing pain experiences organized into four categories: sensory, affective, evaluative, and mixed. The pain assessment index is calculated as the sum of the aggregated values of the words chosen within the subcategories, with the maximum score for each category being as follows: sensory=34, affective=17, evaluative=5, and mixed=11; total=67. The McGill pain questionnaire also contains a scale of pain intensity at the time of application (1=weak, 2=moderate, 3=strong, 4=violent, and 5=unbearable) (31).

### Depression and anxiety

Symptoms of depression and general anxiety were assessed at the beginning and at the end of treatment using the Brazilian versions of the Beck Depression Inventory (BDI) (32) and Visual Analog Anxiety Scale (VAS) (33), respectively. The BDI is a self-assessment tool for depression consisting of a questionnaire with 21 items, with score ranging from 0 to 3 (higher scores indicate more depressive symptoms) (32). The VAS for general anxiety is a horizontal 100-mm-long line. The extreme left end indicates no anxiety, and the extreme right end indicates the worst anxiety possible (33).

These tools were administered before treatment, after three and six weeks, and at three months of follow-up.

### Sample calculation

The sample size was calculated considering the difference between two points on the numerical pain scale (20%) and between the active SWD and placebo, and an estimated standard deviation of three, based on data from a previous study (34). For a significance level of 0.05 and 80% power, it was estimated that 36 participants would be needed, with 18 in each group (Minitab, v.15, USA). The G-Power 3.1 software was used (35).

### Statistical analysis

Descriptive statistics of the variables were performed by calculating the means±SD, and minimum and maximum values for quantitative variables and absolute and relative frequencies for categorical variables. The normality of the variables was verified using the Shapiro-Wilk test. Sociodemographic variables were compared between the groups at the pre-intervention stage using the independent *t*-test and chi-squared test. The effects of time, group, and the interaction between time and group were measured using repeated measures analysis of variance (RM-ANOVA), applying Bonferroni's *post hoc* test for the time factor. Missing data were treated using mean imputation for the intention-to-treat analysis. The statistical software SPSS (Statistical Package for Social Science) version 15.0 for Windows was used (IBM, USA). The significance level adopted was 5%.

## Results

### Demographic characteristics

Of the 36 study participants, 33 completed all the phases. Regarding sociodemographic characteristics, there were no statistically significant differences between the groups at baseline (Table 1).

**Table 1:** Baseline characteristics of the control and intervention groups.

Variable	Intervention (n=18)	Control (n=18)	P-values
Age*	41.17±13.86	39.83±14.42	0.779
Race**			
White	14 (77.77)	15 (83.33)	0.668
Yellow	1 (5.55)	2 (11.11)	
Black	1 (5.55)	1 (5.55)	
Brown	1 (5.55)	0	
Unable to report	1 (5.55)	0	
Gender**			
Female	6 (33.33)	9 (50.00)	0.310
Male	12 (66.66)	9 (50.00)	
Marital status**			
Single	8 (45.00)	9 (50.00)	0.943
Married	9 (50.00)	8 (45.00)	
Divorced	1 (5.00)	1 (5.00)	
Number of children			
None	9 (50.00)	11 (61.11)	
One	2 (11.11)	2 (11.11)	
Two	3 (16.66)	5 (27.77)	
Three	3 (16.66)	0	
Four	1 (5.55)	0	
Schooling**			
Elementary	1 (5.55)	1 (5.55)	0.308
High school	4 (22.22)	1 (5.55)	
Incomplete university	1 (5.55)	4 (22.22)	
Complete university	12 (66.66)	12 (66.66)	
Work status**			
Working	14 (77.77)	14 (77.77)	1.000
Not working	4 (22.22)	4 (22.22)	

*Mean ± SD; independent Student’s *t*-test. **Number (%); chi-squared test.

### McGill Pain Questionnaire

**Figure 2:**
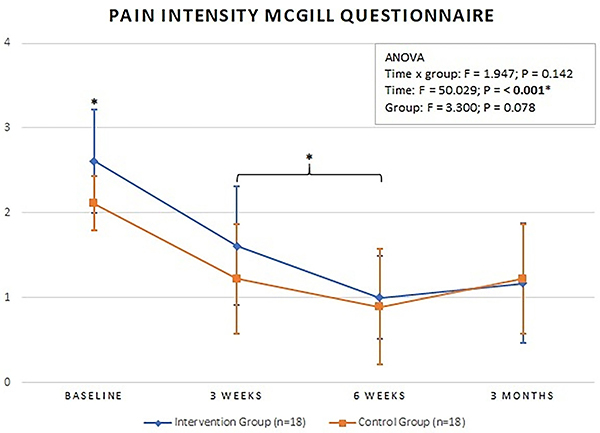
Mean pain intensity values at baseline, 3 weeks, 6 weeks, and 3 months. Intervention Group: completed cases using the intention-to-treat analysis. *P<0.05 for time (ANOVA).

**Figure 3:**
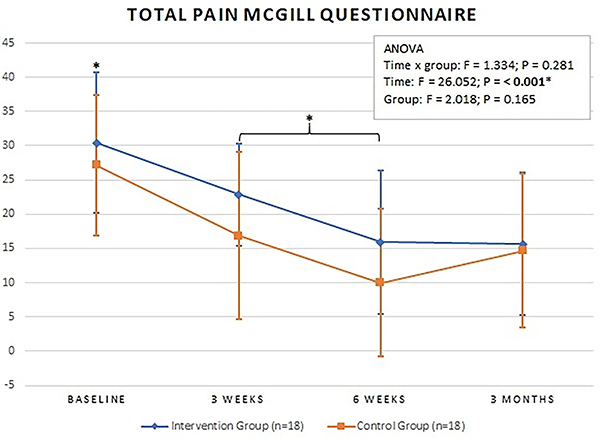
Mean total pain values at baseline, 3 weeks, 6 weeks, and 3 months. Intervention Group: completed cases in intention-to-treat analysis. Data are reported as means±SD. *P<0.05 for time (total pain: sensory, affective, evaluative, and mixed pain aspects); ANOVA.

There was no statistically significant difference between the groups for pain intensity and total pain (sensory, affective, evaluative, and mixed pain aspects) at baseline, three and six weeks, and three months. Both groups had an improvement in these variables throughout the treatment and at the three-month follow-up. (Figures 2 and 3 and Table 2, respectively).

**Table 2:** Mean values for the sensory, affective, evaluative, and mixed aspects of pain at baseline, 3 weeks, 6 weeks, and 3 months for the two groups.

Variable McGill	Intervention				Control				ANOVA		ANOVA	
	Baseline (1)	3 weeks (2)	6 weeks (3)	3 months (4)	Baseline (1)	3 weeks (2)	6 weeks (3)	3 months (4)	Time × group		Time	
									P	F	P*	F
Sensory	15.83±5.63	12.22 ±5.31	8.56±5.80	8.47±5.62	15.22±6.08	9.39±7.01	6.33±7.05	9.11±7.17	0.260	1.404	<0.001	16.079
Affective	7.61±3.33	5.33±2.52	3.69±2.90	3.42±2.61	6.06±3.26	3.72±3.85	1.56±2.04	2.22±1.67	0.384	1.049	<0.001	19.817
Evaluative	2.89±1.02	1.94±0.80	1.36±0.72	1.32±0.71	2.33±0.69	1.61±0.98	1.06±0.87	1.39±0.70	0.268	1.375	<0.001	27.163
Mixed	4.11±2.03	3.39±1.34	2.31±2.09	2.47±2.05	3.56±2.15	2.11±1.75	1.06±1.73	1.94±2.36	0.466	0.871	<0.001	12.198

*Post hoc Tukey test Bonferroni Fit. (Sensory: (1-2) **P=0.001**; CI [1.63 7.81]; (1-3) **P<0.001**; CI [4.81 11.35]; (1-4) **P<0.001**; CI [3.55 9.93]; (2-3) **P=0.002**; CI [1.00 5.72]; (2-4) P=0.215; CI [-0.57 4.60]; (3-4) P=0.669; CI [−3.65 0.96]; Affective: (1-2) **P<0.001**; CI [0.90 3.71]; (1-3) **P<0.001**; CI [2.68 5.73]; (1-4) **P<0.001**; CI [2.49 5.54]; (2-3) **P<0.001**; CI [0.80 3.00]; (2-4) **P=0.008**; CI [0.34 3.08]; (3-4) P=1.000; CI [-0.99 0.60]; Evaluative: (1-2) **P<0.001**; CI [0.35 1.32]; (1-3) **P<0.001**; CI [0.96 1.85]; (1-4) **P<0.001**; CI [0.81 1.70]; (2-3) **P=0.003**; CI [0.15 0.99]; (2-4) P=0.106; CI [-0.05 0.90]; (3-4) P=1.000; CI [-0.51 0.22]; Mixed: (1-2) **P<0.035**; CI [0.51 2.12]; (1-3) **P<0.001**; CI [1.07 3.24]; (1-4) **P=0.001**; CI [0.52 2.74]; (2-3) **P<0.001**; CI [0.42 1.72]; (2-4) P=0.522; CI [-0.32 1.40]; (3-4) P=0.207; CI [-1.20 0.14]. CI: Confidence Interval (95%).

### Beck Depression Inventory

**Figure 4:**
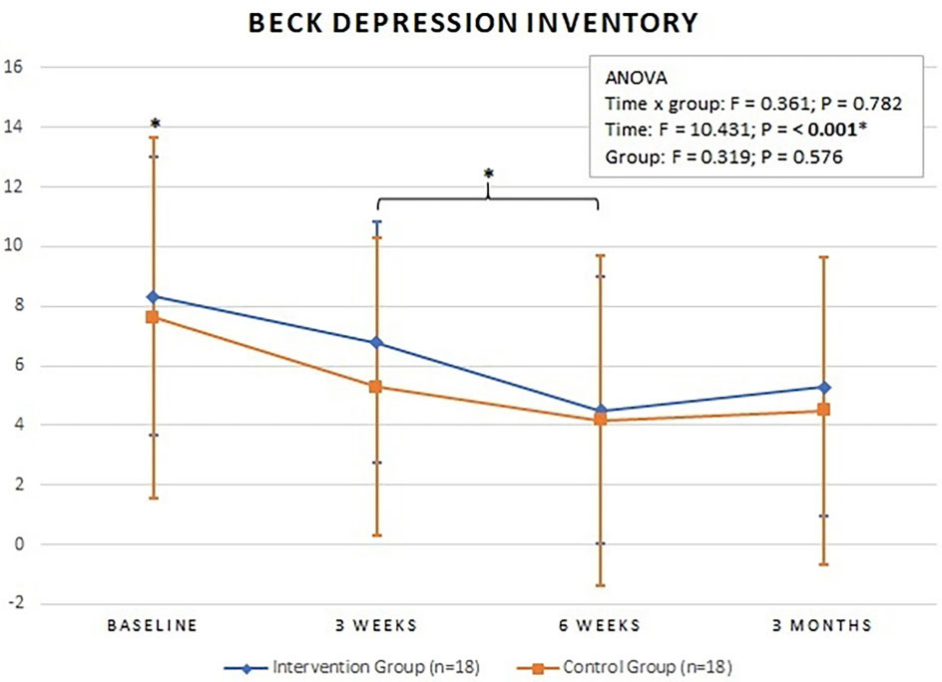
Mean values of the Beck Depression Inventory at baseline, 3 weeks, 6 weeks, and 3 months. Intervention Group: completed cases using the intention-to-treat analysis. Data are reported as means±SD. *P<0.05 for time (ANOVA).

There was no statistically significant difference in depressive symptoms between the groups at all time points. Both interventions showed improvement between the beginning and end of follow-up (Figure 4).

### Visual Analog Anxiety Scale

**Figure 5:**
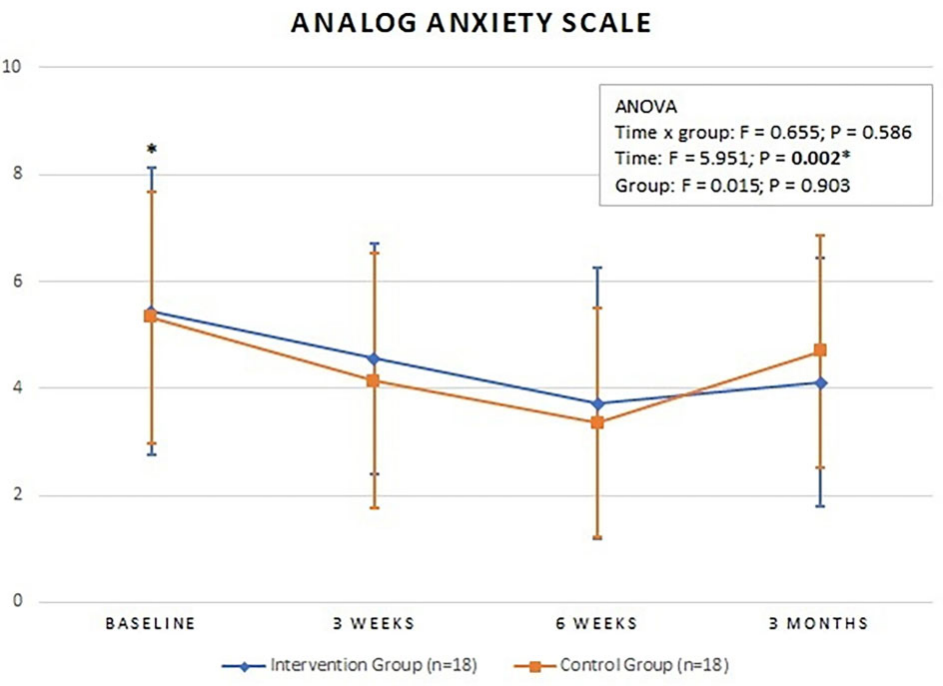
Mean values of the Visual Analog Anxiety Scale at baseline, 3 weeks, 6 weeks, and 3 months. Intervention Group: completed cases using the intention-to-treat analysis. Data are reported as means±SD. *P<0.05 for time (ANOVA).

There was also no statistically significant difference between the groups at all time points. Both groups improved during treatment, but there was no significant difference between baseline and three-month follow-up (Figure 5).

## Discussion

This study aimed to evaluate the effect of the association between CSWD and Pilates on reducing pain, depression, and anxiety in individuals with CNLBP. However, we found no statistically significant difference between the evaluated groups, and both groups improved at follow-up, with the exception of the anxiety variable, which returned to pre-treatment levels in this assessment.

Our study assumed that the association between Pilates and a form of vigorous heat (CSWD) could benefit patients in aspects related to pain, depression, and anxiety, as intense heat activates TRPV1 receptors, which are present in the brain regions involved in emotions, such as depression and anxiety.

The role of heat in improving depressive symptoms is not yet fully understood, but studies on capsaicin, which also stimulates TRPV1 receptors, have shown that it acts as an agonist of the antidepressant effects associated with amitriptyline (23). Lin et al. (36) pointed out that hyperalgesia and depressive behavior were associated with a decrease in the activity of the TRPV1 signaling pathway in the medial prefrontal cortex, hippocampus, and periaqueductal gray of mice with comorbid chronic pain and depression. Furthermore, they found that electroacupuncture activated TRPV1 receptors, showing improvement in depressive symptoms. As heat also activates TRPV1 receptors, an increase in its activation could contribute to the improvement of depressive symptoms, but we were unable to observe this effect in this study.

As the analyzed variables did not show additional improvement in CNLBP treatment with the use of CSWD, it seems that performing Pilates is sufficient to control pain, depression, and anxiety. Karasel et al. (37) found similar results in terms of pain and depression, which improved in the intragroup assessment in both the intervention and control groups. However, there were no significant differences between the groups.

Anxiety symptoms are another important aspect to be improved in patients with CNLBP. Hu et al. (38) recommended that health professionals pay more attention to anxiety symptoms of patients with CNLBP with low income, long duration of pain, severe pain, poor family functioning, and low pain self-efficacy.

Our study also revealed that the application of CSWD had no significant effect on anxiety improvement. Additionally, we found that patients in both groups had moderate anxiety at the beginning of treatment, which changed to mild anxiety at the sixth week of treatment. After the 3-month follow-up, both groups returned to moderate anxiety levels, demonstrating that they managed to reduce anxiety levels only while receiving treatment.

In a systematic review, Chong et al. (39) affirmed that there is limited evidence that exercise interventions have the potential to be effective, feasible, and safe non-pharmacological interventions for anxiety disorders in mid- and late life.

Finally, our study corroborates the systematic review conducted by Lemieux et al. (40), which found no statistically significant differences in disability, quality of life, and pain between exercise and other non-pharmacological interventions in patients with CNLBP. Therefore, to date, exercise programs are sufficient to control this dysfunction.

### Clinical implications

Based on the results of this study, it is suggested that a Pilates-based exercise program is sufficient to treat patients with CNLBP, without the need for the addition of CSWD.

### Study limitations

Due to the nature of this clinical intervention, there was no possibility of blinding the therapist for group allocation, given the thermal effect of CSWD. The study design did not allow for assessments by age group, which is a limitation of the study.

### Conclusion

It was concluded that active CSWD did not provide additional improvements in pain, depression, and anxiety variables in patients with CNLBP. Both groups improved pain and depression at follow-up and reduced anxiety only during Pilates exercises, thus, proving that they are sufficient for treating this condition.
